# Notes and News

**Published:** 1896-12-26

**Authors:** 


### Notes and News.

(Collected and Communicated.)

A gentleman has promised a contribution of £500 to University College Hospital, provided that nine others will promise the same amount, or £100 per annum for five years.

It is decided to build a general hospital at Belfast to commemorate the sixty years of the Queen's reign. The new institution will be called the Victoria Hospital, and £100,000 will be required for its erection.

The Duke of Fife will preside at the triennial festival of the Homes for Working Boys in London, at St. Martin's Town Hall, on the evening of Wednesday, January 20th, when a dinner will be given to the 320 boys now residing in the seven homes.

We regret that the two following institutions were omitted from the list of those under the patronage of the Queen in our Christmas Appeal Supplement: The Royal Albert Asylum for Idiots and Imbeciles of the Northern Counties, founded in 1864, and the Royal Society of Musicians of Great Britain, founded in 1738.

Dr. Thorne Thorne, chief medical officer of the Local Government Board, accompanied by Dr. Copeman, is visiting various places on the Continent with the object of studying the systems of vaccination and the laws and regulations bearing on the subject. He has just returned from Brussels. One of the main objects of his inquiries is understood to be as to the efficacy of the glycerine preparations of lymph advocated by Dr. Copeman.

The proposal to amalgamate the two eye hospitals in Dublin, the St. Mark's Ophthalmic and the Aural Hospital and the National Eye and Ear Infirmary, have advanced so far that at a meeting of the conjoint committee, held on December 8th, the draft Act of Parliament necessary to effect this amalgamation was formed, and after a final revision was adopted. A petition to the House of Commons on the subject was signed by the members of the committee.

Mr. Thomas Bryant, F.R.C.S., has been appointed to the post of Honorary Consulting Surgeon to the Hospital for Epilepsy and Paralysis, Regent's Park, rendered vacant by the death of Sir John Eric Erichsen, Bart.

The result of the election for direct representatives on the General Medical Council was as follows: For England—Robert Reid Rentoul, 6,646 votes; George Brown, 5,369; James Grey Glover, 4,910. For Scotland —William Bruce, 1,311.

The Parke Memorial statue in Leinster Lawn, Dublin, was unveiled on December 19th by Field Marshal Lord Roberts before a large assembly. Among the letters of apology for non-attendance was one from Mr. H. M. Stanley, referring in eulogistic terms to the late Surgeon-Major Parke.

The Prince of Wales, President of the St. John Ambulance Association, has sanctioned the holding of a fête and ambulance demonstration at the Crystal Palace in commemoration of Her Majesty's long reign. The fête will be under the patronage of Princess Christian and other members of the Royal Family, and will take place in May or June next.

The William Gosage Laboratories and other extensions of the chemical laboratories of University College, Liverpool, were opened on Saturday, December 12th, by the Earl of Derby, in the presence of a large and influential gathering, including the Lord Mayor of Liverpool. The buildings include a large laboratory fitted up for forty-four advanced students, and an additional lecture-room, together with a metallurgical laboratory and other additional rooms.

The authorities of the Coventry Hospital have got into difficulties in regard to the election of a member of the honorary medical staff by adopting the course, always an injudicious one, of altering the rules as to the qualifications at the moment when an election is imminent. As luck would have it, not only have they incurred the odium attaching to this course, but they have deprived the hospital of any benefit which might have accrued to it from their action by resolving that the reform shall not take effect until after the coming election.

Those who are interested in the introduction of women into the medical profession are anxious to improve and extend the premises in which the medical education of women is at present carried on in London, and to obtain increased support for the New Hospital for Women, the only hospital of the sort in London which is entirely officered by qualified women. At present the school is carried on in houses in Handel Street and Hunter Street, which are hired for the purpose, but it is proposed to pull these down and build good laboratories, library, and lecture-rooms, on ground for which a long lease is being arranged with the governors of the Foundling Hospital. To avoid interruption of the work of the school. the alterations will have to be carried on gradually, but it is proposed to put up a large block of laboratories immediately, so as to have them ready for October, 1897. The entry of students last October was so large that it is already found very difficult to provide places for them in the practical classes. The cost of the whole scheme is estimated at about £20,000. The New Hospital for Women also asks for money.

